# Integrative genomics reveal a role for MCPIP1 in adipogenesis and adipocyte metabolism

**DOI:** 10.1007/s00018-019-03434-5

**Published:** 2019-12-31

**Authors:** Magdalena Losko, Dobrochna Dolicka, Natalia Pydyn, Urszula Jankowska, Sylwia Kedracka-Krok, Maria Kulecka, Agnieszka Paziewska, Michal Mikula, Piotr Major, Marek Winiarski, Andrzej Budzynski, Jolanta Jura

**Affiliations:** 1 Department of General Biochemistry, Faculty of Biochemistry, Biophysics and Biotechnology, Jagiellonian University, Gronostajowa 7, 30-387 Kraków, Poland; 2 Proteomics and Mass Spectrometry Laboratory, Malopolska Centre of Biotechnology, Jagiellonian University, Gronostajowa 7A, 30-387 Kraków, Poland; 3 Department of Physical Biochemistry, Faculty of Biochemistry, Biophysics and Biotechnology, Jagiellonian University, Gronostajowa 7, 30-387 Kraków, Poland; 4 Department of Gastroenterology, Hepatology and Clinical Oncology, Medical Center for Postgraduate Education, Marymoncka 99/103, 01-813 Warsaw, Poland; 5 Department of Genetics, Maria Sklodowska-Curie Memorial Cancer Center and Institute of Oncology, Wawelska 15B, 02-034 Warsaw, Poland; 6 Second Department of General Surgery, Centre for Research, Training and Innovation in Surgery (CERTAIN Surgery), Jagiellonian University Medical College, Kopernika 21, 31-501 Kraków, Poland

**Keywords:** Regnase-1, Pparγ, C/EBPα, Lipid homeostasis

## Abstract

Obesity is considered a serious chronic disease, associated with an increased risk of developing cardiovascular diseases, non-alcoholic fatty liver disease and type 2 diabetes. Monocyte chemoattractant protein-1-induced protein-1 (MCPIP1) is an RNase decreasing stability of transcripts coding for inflammation-related proteins. In addition, MCPIP1 plays an important role in the regulation of adipogenesis in vitro by reducing the expression of key transcription factors, including C/EBPβ. To elucidate the role of MCPIP1 in adipocyte biology, we performed RNA-Seq and proteome analysis in 3T3-L1 adipocytes overexpressing wild-type (_WT_MCPIP1) and the mutant form of MCPIP1 protein (_D141N_MCPIP1). Our RNA-Seq analysis followed by confirmatory Q-RT-PCR revealed that elevated MCPIP1 levels in 3T3-L1 adipocytes upregulated transcripts encoding proteins involved in signal transmission and cellular remodeling and downregulated transcripts of factors involved in metabolism. These data are consistent with our proteomic analysis, which showed that MCPIP1 expressing adipocytes exhibit upregulation of proteins involved in cellular organization and movement and decreased levels of proteins involved in lipid and carbohydrate metabolism. Moreover, MCPIP1 adipocytes are characterized by decreased level of insulin receptor, reduced insulin-induced Akt phosphorylation, as well as depleted Glut4 level and impaired glucose uptake. Overexpression of Glut4 in 3T3-L1 cells expressed _WT_MCPIP1 rescued adipogenesis. Interestingly, we found decreased level of MCPIP1 along with an increase in body mass index in subcutaneous adipose tissue. The presented data show a novel role of MCPIP1 in modulating insulin sensitivity in adipocytes. Overall, our findings demonstrate that MCPIP1 is an important regulator of adipogenesis and adipocyte metabolism.

**Electronic supplementary material:**

The online version of this article (10.1007/s00018-019-03434-5) contains supplementary material, which is available to authorized users.

## Introduction

Monocyte chemoattractant protein-1-induced protein-1 (MCPIP1), also known as Regnase-1, is an RNase controlling stability of many transcripts and miRNAs. The ribonucleolytic activity of MCPIP1 is associated with the PIN domain—named after its identification in the N-terminus of the PilT protein (PilT N-terminus). Another domain essential for effective cleavage of RNA molecules is the zinc finger domain. MCPIP1 cleaves its targets binding first to stem loop sequences present near the Stop codon in the 3′ untranslated region of the transcript [1, 2]. In the case of miRNA, MCPIP1 degrades precursor transcripts, effectively terminating microRNA biogenesis [3].

Although the complete list of MCPIP1 targets remains unknown, directly targeted transcripts identified thus far suggest that MCPIP1 is involved in many cellular processes. It was shown that MCPIP1 regulates inflammatory processes by controlling transcripts coding for proinflammatory cytokines, such as IL-1beta, IL-6, IL-12p40, IL-2 and transcription factors controlling proinflammatory processes, such as the c-Rel subunit of NFκB [4–7]. Furthermore, MCPIP1 regulates processes important in cancer development, such as cell metabolism, proliferation, and angiogenesis [8–10].

There is a growing body of evidence showing that MCPIP1 also plays a critical role in cell differentiation. Although the exact mechanism of this process is not yet clear, the literature suggests MCPIP1 plays an important role in differentiation of neuronal progenitor cells, monocytes, and preadipocytes [11–14]. Differentiation of preadipocytes towards adipocytes, called adipogenesis, is an important process tightly controlled by communication between individual cells and with the extracellular environment. Impaired differentiation of adipocyte precursors is a consequence of enhanced inflammation and dysregulation of signaling pathways in the cells of fat tissue. Interestingly, it was shown that hypertrophic obesity is associated with diminished recruitment and differentiation of preadipocytes.

Experiments by Lipert and colleagues using 3T3-L1 preadipocytes showed that MCPIP1 plays a pivotal role during adipogenesis [13]. This protein is involved in degradation of the transcript encoding CCAAT/Enhancer Binding Protein Beta (CEBPβ), one of the major transcription factors responsible for differentiation of preadipocytes into adipocytes [13, 15]. Furthermore, using Next-Generation Sequencing (NGS) Losko et al. demonstrated that MCPIP1 overexpression modulates levels of 58 miRNAs on day 2 of adipocyte differentiation [14]. Changes in these miRNA levels influences 19 signaling pathways that are important for adipogenesis.

To further clarify the role of MCPIP1 in adipogenesis and adipocyte metabolism we analyzed this protein in human adipose tissue taken from lean and obese subjects. Furthermore, we conducted a time course analysis of transcript and protein profiles in 3T3-L1 cells expressing wild-type MCPIP1 (_WT_MCPIP1) or mutant MCPIP1 (_D141N_MCPIP1) during adipocyte differentiation.

## Materials and methods

### Patient tissue samples

Adipose tissue samples (subcutaneous and visceral) were obtained from patients treated laparoscopically due to morbid obesity or inguinal hernia (control group) in The Second Department of General Surgery, Jagiellonian University Medical College (Krakow, Poland). Criteria for surgical treatment in morbid obesity patients were in accordance with the guidelines of the Metabolic and Bariatric Surgery Section of the Polish Surgical Society (i.e., BMI ≥ 35 kg/m^2^ with obesity comorbidities or BMI ≥ 40 kg/m^2^). All bariatric patients [eight men, eleven women; mean age 44 (11.0) years (range 24–64); mean BMI—47.5 (7.6) kg/m^2^] had undergone laparoscopic sleeve gastrectomy (SG). This group also included patients with type 2 diabetes—11 individuals. Patients with inguinal hernia [control group, six men, three women; mean age 44 (15.0) years (range 21–66); mean BMI—24.7 (3.6) kg/m^2^] were qualified for treatment according to The European Hernia Society recommendation. This group also included 1 patient with type 2 diabetes. In this group surgeons performed elective inguinal hernia repair using transabdominal preperitoneal approach (TAPP) [16, 17]. Patients from both groups were treated in accordance with the principles of the multimodal Enhanced Recovery After Surgery (ERAS) pathway. Exclusion criteria included occurrence of perioperative complications, history of cancer, severe liver disease, coagulopathy, and smoking or alcohol abuse [18]. The characteristic of patients was summarized in supplementary data (Table S1).

All human tissue samples were collected according to the established protocol approved by the Local Ethics Committee (approval no. 1072.6120.119.2017). Visceral fat samples were collected from the greater omentum using high-energy devices—LigaSure™ (Covidien/Medtronic, Mansfield, MA, USA) and THUNDERBEAT™ (Olympus, Shinjuku, Japan). Small fragments of the subcutaneous, preperitoneal fat tissue from the umbilical region were excised with scissors and bipolar coagulation. Samples were used for RNA and protein isolation (frozen in liquid nitrogen and stored at − 80 °C).

### Cell culture

The 3T3-L1 cell line was purchased from the American Type Culture Collection (Manassas, VA, USA). 3T3-L1 fibroblasts were cultured in Dulbecco’s modified Eagle’s medium (DMEM) with 4.5 g/l glucose (Lonza, Basel, Switzerland) supplemented with 10% bovine calf serum (Biowest, Nuaillé, France) at 37 °C in a humidified, 5% CO_2_ atmosphere. Culture density was not allowed to exceed 70% confluence.

### Transduction of preadipocytes with retroviral vectors

The pMX retrovirus system was used to express MCPIP1 or Glut4 in 3T3-L1 adipocytes. Vectors include pMX-MCPIP1 (encoding wild-type human MCPIP1; named _WT_MCPIP1), pMX-MCPIP1-D141N (encoding human MCPIP1 with a single point mutation within the region of an enzymatically active PIN domain; named _D141N_MCPIP1), pMX-Glut4 (encoding wild-type murine Glut4; named Glut4) and pMX-PURO (control cells with an empty vector; named Control). Viral titer estimation prior to transduction of preadipocytes was performed according to a previously described procedure [13, 14]. After 24 h of incubation medium was changed and 48 h post-transduction cells were cultured in complete growth medium supplemented with 2 μg/ml puromycin (Invivogen, San Diego, CA, USA) for the next 4–5 days. For the rescue experiments, 3T3-L1 cells with both _WT_MCPIP1 and Glut4 expression were generated. For this purpose, 3T3-L1 preadipocytes transduced with pMX-MCPIP1 vector, following by selection with puromycin, were infected for 24 h with the pMX-Glut4 vector in complete medium containing 6 μg/ml polybrene (Millipore, Burlington, MA, USA). After transduction, the infection medium was replaced with fresh complete growth medium and cells were selected with puromycin (2 μg/ml) for another 4 days. Cells were then used for appropriate experiments as described below.

### Differentiation of preadipocytes

For differentiation, 7 × 10^4^/cm^2^ 3T3-L1 preadipocytes transduced with retroviral vectors (_WT_MCPIP1, _D141N_MCPIP1, Glut4, _WT_MCPIP1 + Glut4 and control cells) were seeded on a culture plate, and 48 h after reaching confluence, the cells were exposed to a differentiation medium (DMI medium), containing 5 μg/ml insulin (Eli Lilly, Indianapolis, IN, USA), 0.25 μM dexamethasone (Sigma Aldrich, Saint Louis, MO, USA) and 0.5 mM isobutylmethylxanthine (Sigma Aldrich) dissolved in DMEM containing 10% FBS. Two days later, the medium was switched to DMEM with 10% FBS and 10 μg/ml insulin. The medium was replaced every 2 days until day 8.

### RNA sequencing

#### RNA extraction

Total RNA was extracted from tissue using mirVana™ PARIS™ Kit (Thermo Fisher Scientific, Waltham, MA, USA) according to manufacturer’s instructions. The concentration of each sample was measured using a NanoDrop 2000 (Thermo Fisher Scientific). Total RNA quality was analyzed with the RNA 6000 Nano Kit on Agilent 2100 Bioanalyzer (Agilent, Santa Clara, CA, USA). Only samples with an RNA integrity number (RIN) > 7 were considered for downstream analyses.

#### Poly(A) fraction purification and sequencing

Messenger RNA (poly(A)-containing mRNA) was purified from 1–8 μg of total RNA by magnetic beads coated with oligo (dT) using a Dynabeads^®^ mRNA DIRECT™ Micro Kit (Thermo Fisher Scientific). The quality and quantity of mRNA samples was analyzed with the RNA 6000 Pico Kit with the 2100 Bioanalyzer (Agilent). The sequencing library of each RNA sample was prepared using Ion Total RNA-Seq Kit v2 according manufacturer’s protocol (Thermo Fisher Scientific). The libraries were prepared from 1 to 15 ng of mRNA. Briefly, the mRNA was fragmented using RNaseIII and then purified. The fragmented RNA was hybridized and ligated with Ion adaptors. The RNA fragments were then reverse transcribed and amplified to double-stranded cDNA. Next, the cDNA was purified by magnetic bead based method. The molar concentration and size selection (50–1000 bp) of each cDNA library was determined using DNA 1000 Kit on Bioanalyzer 2100 (Agilent). Each library was diluted to ~ 80 pM prior to template preparation and up to four barcoded libraries were mixed in equal volume and used for automatic template preparation on an Ion Chef (Thermo Fisher Scientific) instrument using reagents from the Ion PI Hi-Q 200 Kit (Thermo Fisher Scientific) and Ion PI v3 Proton Chip. Samples were sequenced on the Ion Proton System (Thermo Fisher Scientific) according to the manufacturer’s instructions.

#### Gene expression analysis

Raw reads were mapped to mm10 reference genome with Ion Torrent RNASeqAnalysis Plugin version 5.0 which utilizes STAR as the primary aligner, and bowtie2 for previously unaligned reads [19, 20]. Gene abundance was quantified with htseq-count (HTSeq framework version 0.6) using Ensembl Gene gtf file from UCSC as reference [21]. Differential gene expression was performed with R package DESeq2 version 1.10.1. Gene expression data has been deposited in the European Nucleotide Archive, entry PRJEB27195. Additionally, a complete list of the expression levels of each gene with the statistical results, studied in _WT_MCPIP1 vs Control and _WT_MCPIP1 vs. _D141N_MCPIP1 group was presented in supplementary data (Table S2).

### Bioinformatics analysis of RNA-sequencing data

Functional annotation of differentially expressed genes (≥ 1.5-fold of change) was performed online using bioinformatic tools available via the Database for Annotation, Visualization and Integrated Discovery (DAVID; version 6.8; https://david.ncifcrf.gov/home.jsp). The default search parameters were used. Gene lists were searched using Ensembl gene annotation (ENSEMBL_GENE_ID). The background dataset used for analyses was the *Mus musculus* genome. Selected genes were classified into functional-related gene groups using the Biological Process (BP) GO category and KEGG Pathways.

### RNA isolation and Q-RT-PCR analysis

Total RNA from cultured cells and tissues was isolated using Fenozol (A&A Biotechnology, Gdynia, Poland) according to the manufacturer’s instructions. The concentration and purity of isolated RNA was quantified spectrophotometrically using ND-1000 spectrophotometer (Thermo Fisher Scientific). Total RNA (1 μg) was reverse transcribed into complementary cDNA, using an oligo(dT) primer and M-MLV reverse transcriptase (Promega, Madison, WI, USA). Following synthesis, cDNA was diluted 5 times and subjected to qRT-PCR amplification with a SYBR Green PCR master mix (A&A Biotechnology) and gene-specific primer pairs. Values for mRNA expression were normalized to the level of eukaryotic translation elongation factor 2 expression. For the 3T3-L1 cells, the relative expression values are presented as fold changes in comparison to Control group. The sequences of the primers are presented in the supplementary data (Table S3).

### Mass spectrometric analysis of MCPIP1-associated proteins

#### Protein extraction and digestion

The protein lysates were collected in three biological replicates from adipocytes at day 4 of differentiation. Cells were stably transduced with retroviral vectors encoding wild-type MCPIP1 (_WT_MCPIP1), mutant MCPIP1 (_D141N_MCPIP1), or an empty vector control. The samples were processed using the filter-aided sample preparation (FASP) method with some modifications [22]. Briefly, 100 µg of each sample was brought to 300 µl with a urea solution (8 M urea, 50 mM ammonium bicarbonate) and reduced with 50 mM dithiothreitol (DTT) for 15 min at 37 °C. Samples were centrifugated at 20,000×*g* for 10 min at 20 °C and the supernatant was loaded on centrifugal concentrator with a 30-kDa membrane cut-off (Vivacon 500, Sartorius Stedim Biotech GmbH). SDS buffer was replaced with urea solution and proteins were alkylated with 50 mM iodoacetamide for 20 min. Next, samples were washed three times with urea solution and three times with 50 mM ammonium bicarbonate. Proteins were digested for 16 h with trypsin (Promega) at an enzyme to protein ratio of 1:50. The resulting peptides were collected by centrifugation and washed out with 0.5 M NaCl and 50 mM ammonium bicarbonate. The peptides were acidified with trifluoroacetic acid (TFA).

#### Liquid chromatography and tandem mass spectrometry (LC–MS/MS)

The nanoLC–MS/MS analysis was performed in duplicate on a Q-Exactive mass spectrometer (Thermo Fisher Scientific) coupled with a nanoHPLC (UltiMate 3000 RSLCnano System, Thermo Fisher Scientific). The peptide samples were loaded onto a trap column (Acclaim PepMap 100 C18, 75 μm × 20 mm, 3 μm particle, 100 Å pore size, Thermo Fisher Scientific) in 2% acetonitrile with 0.05% TFA at a flow rate of 5 μl/min and further separated on analytical column (Acclaim PepMap RSLC C18, 75 µm × 500 mm, 2 µm particle, 100 Å pore size, Thermo Fisher Scientific) with a 240 min gradient from 2 to 40% acetonitrile in 0.05% formic acid at a flow rate of 300 nl/min. Eluting peptides were ionised using a Digital PicoView 550 nanospray source (New Objective, Woburn, MA, USA) and acquired in an MS data dependent mode using the top twelve method with 30 s of dynamic exclusion. Full scan MS spectra were acquired with a resolution of 70,000 over a mass range of 300 to 2000 *m*/*z* with an automatic gain control (AGC) target of 1e6. The MS/MS spectra were acquired with a resolution of 17,500 with an AGC target of 5e5. The maximum ion accumulation times for the full MS and the MS/MS scans were 120 ms and 60 ms, respectively. The lock mass option was used to perform internal calibration.

#### Data processing

Raw MS data files were processed using MaxQuant software (version 1.5.5.1) [23]. Peak lists were searched against the forward and reverse Swissprot_201608 database (*Mus musculus*, 16 813 sequences) using integrated Andromeda search engine [24]. Only fully tryptic peptides with up to two missed cleavages and minimum length of seven amino acids were considered. Cysteine carbamidomethylation was set as a fixed modification and methionine oxidation as a variable modification. The precursor mass tolerance in the first search used for mass recalibration was set to 20 ppm. The main search was performed with precursor and fragment mass tolerance 4.5 ppm and 20 ppm, respectively. The match between runs option was enabled within a time window of 0.7 min. The maximum false discovery rate (FDR) for both peptide and protein identifications was set to 0.01. All proteins that could not be distinguished based on the identified peptides were merged to one protein group. Relative quantification and normalization was performed with the MaxLFQ label-free algorithm using a minimum ratio count of 2. Both razor and unique peptides were used for protein quantitation [25].

#### Bioinformatic and statistical analysis

Analyses were performed with the Perseus software (version 1.5.5.3) [26]. Contaminants, proteins from the reverse database and proteins identified only with modified peptides were excluded from the study. All bioinformatic analyses were executed on LFQ intensities transformed to logarithmic scale with base two. Proteins identified in all three biological replicates in one group, and not present in any replicate in the second group were considered as qualitative differences between compared cell lines. To identify significant expression changes at the protein level, a two-sample Student’s *t* test was applied with the S0 parameter set to 1, followed by a permutation-based false discovery rate (FDR) correction for multiple hypothesis testing at a 0.01 threshold. Only proteins with valid values in each biological replicate, in both compared groups, were taken into consideration. In the comparison between _D141N_MCPIP1 and control no proteins met these thresholds, therefore in this case less restrictive criteria were used, namely: S0 set to 0.2 and FDR set to 0.05. Original mass spectrometry data have been deposited to the ProteomeXchange Consortium via the PRIDE partner repository with the dataset identifier PXD010148 [27].

#### Pathway analysis

Qualitative and quantitative differences were uploaded to the Ingenuity Pathway Analysis software (IPA, QIAGEN, Redwood City, CA, USA) and KEGG Pathways (DAVID; version 6.8; https://david.ncifcrf.gov/home.jsp) to gain insight into biological processes and pathways that are preferentially altered in compared cell lines. Fischer’s exact test was used to determine the probability that the association between the proteins in the data set and the canonical pathway is explained by chance alone. The *z*-score was calculated to evaluate the match of observed and predicted up/down regulation patterns [28].

### Immunofluorescence staining

For immunofluorescence staining, 3T3-L1 cells stably transduced with retroviral vectors (_WT_MCPIP1, _D141N_MCPIP1 and control cells) were seeded on 12 mm glass coverslips in 24-well plates and grown to full confluence. Then cells were stimulated with adipocyte differentiation medium as described above. At day 8 of differentiation, cells were fixed with 4% paraformaldehyde (PFA) for 15 min at room temperature, washed 3 times with PBS and permeabilized in PBS with 1% Triton X-100 (Bioshop, Burlington, Canada) for 20 min. Next cells were blocked for 1 h with 1% BSA in PBS with 0.2% Triton X-100. The cells were immunostained with mouse monoclonal anti-GLUT4 antibody (1:250 dilution; Thermo Fisher Scientific) overnight at 4 °C followed by incubation with goat anti-mouse IgG antibody conjugated with Alexa Fluor 488 dye (1:800 dilution; Thermo Fisher Scientific) for 1 h at room temperature. All antibodies were diluted in 1% BSA in PBS. Hoechst 33,258 staining was employed as a counterstain for fluorescence (1:10,000 dilution; Thermo Fisher Scientific). Between antibodies incubations, slides were washed with PBS, 3 times per 5 min. As a control, we conducted secondary antibody staining alone under the same staining and exposure conditions. The coverslips were mounted using Fluorescence mounting medium (DakoCytomation, Denmark, UK). Slides were examined using a Leica DMI600B inverted widefield fluorescence microscope (Leica Microsystems, Wetzlar, Germany). All images were taken using 40 × oil immersion objective with Leica LAS X image acquisition software.

### Glucose uptake assay

Adipocyte glucose uptake was measured using the Glucose Uptake-Glo Assay (Promega) according to the manufacturer’s protocol. In brief, 3T3-L1 cells, stably transduced with retroviral vectors (_WT_MCPIP1, _D141N_MCPIP1 and control cells), were seeded in a 96-well white plate at the density of 2 × 10^3^ cells per well. After reaching confluence, adipocyte differentiation was induced with DMI medium. At day 7 of differentiation, cells were starved overnight in serum-free medium (DMEM with 4.5 g/l glucose). Next, cells were starved for glucose by preincubating with 2% bovine albumin serum (BSA; Bioshop) in Krebs-Ringer-Phosphate-HEPES (KRPH; 20 mM HEPES, 5 mM KH_2_PO_4_, 1 mM MgSO_4_, 1 mM CaCl_2_, 136 mM NaCl, 4.7 mM KCl, pH 7.4) buffer for 40 min. Then, cells were stimulated with 1 μM insulin for 20 min, following by 1 mM 2-deoxyglucose (2-DG) incubation for 20 min. 2DG6P Detection Reagent was incubated for 1 h at room temperature. To measure glucose uptake, the luminescence was recorded with 1000 ms integration time using Tecan Spectra Fluor Plus Microplate Reader (Thermo Fisher Scientific) with Tecan iControl 1.5. Software.

### Experimental treatments

To determine the effect of MCPIP1 in insulin signaling pathway, 3T3-L1 adipocytes stably transduced with retroviral vectors (_WT_MCPIP1, _D141N_MCPIP1 and control cells), at day 8 of differentiation, were serum-starved for 2 h, following by stimulation with insulin (0, 1, 10, 100 nM) for 15 min. After incubation, whole protein lysates were subjected to Western blot analysis to measure the levels of the associated insulin signaling proteins.

### Western blot analysis

Cells and adipose tissue samples were lysed in whole cell lysis buffer (62.5 mM Tris–HCl, pH 6.8; 2% SDS; 25% glycerol; 5% β-mercaptoethanol) supplemented with Complete Protease Inhibitor Cocktail (Roche, Basel, Switzerland) and PhosSTOP Phosphatase Inhibitor Cocktail (Roche). Then, 30 μg of the protein lysate was separated on a 10% SDS-PAGE gel and transferred to a PVDF membrane (Merck Millipore, Burlington, ME, USA). The membrane was blocked for 1 h with 5% BSA and incubated with primary antibody overnight at 4 °C. After incubation with HRP-conjugated secondary antibody (1 h at room temperature), chemiluminescence was visualized using Luminata Crescendo (Millipore) substrate in a MicroChemi chemiluminescence detector (Bio-Rad). The following antibodies were used: rabbit anti-p-IGF1R^Tyr1135/1136^/IR^Tyr1150/1151^ (1:1000; Cell Signaling, Danvers, MA, USA), rabbit anti-IR (1:1000; Cell Signaling), rabbit anti-p-Akt^Thr308^ (1:1000; Cell Signaling), anti-p-Akt^Ser473^ (1:1000; Cell Signaling), rabbit anti-PPARγ (1:1000; Cell Signaling), rabbit anti-C/EBPα (1:1000; Cell Signaling), rabbit anti-MCPIP1 (1:1000; GeneTex, Irvine, CA, USA), rabbit anti-MCPIP1 (1:2000; own production), mouse anti-GLUT4 (1:1000; Thermo Fisher Scientific), mouse anti-α-tubulin (1:1000, Calbiochem; Merck Millipore), mouse anti-β-actin (1:4000; Sigma Aldrich), horseradish peroxidase-conjugated anti-rabbit (1:3000; Cell Signaling) and horseradish peroxidase-conjugated anti-mouse (1:20,000; BD Pharmingen, San Diego, CA, USA; or for anti-Glut4: 1:10,000; Cell Signaling).

### Statistical analysis

Data were presented as mean ± standard deviation (SD). Statistical significance was calculated with two-tailed unpaired Student *t* test (for comparison of two groups), one-way ANOVA, two-way ANOVA and Pearson correlation coefficient (for correlation analyses). Statistical analyses were performed using GraphPad Prism (ver. 6.0). *p* values of less than 0.05 were considered significant. *p < 0.05, **p < 0.01, ***p < 0.001 were indicated.

## Results

### MCPIP1 level is decreased in the adipose tissue of obese subjects and negatively correlates with proinflammatory cytokines expression in this tissue

**Fig. 1:**
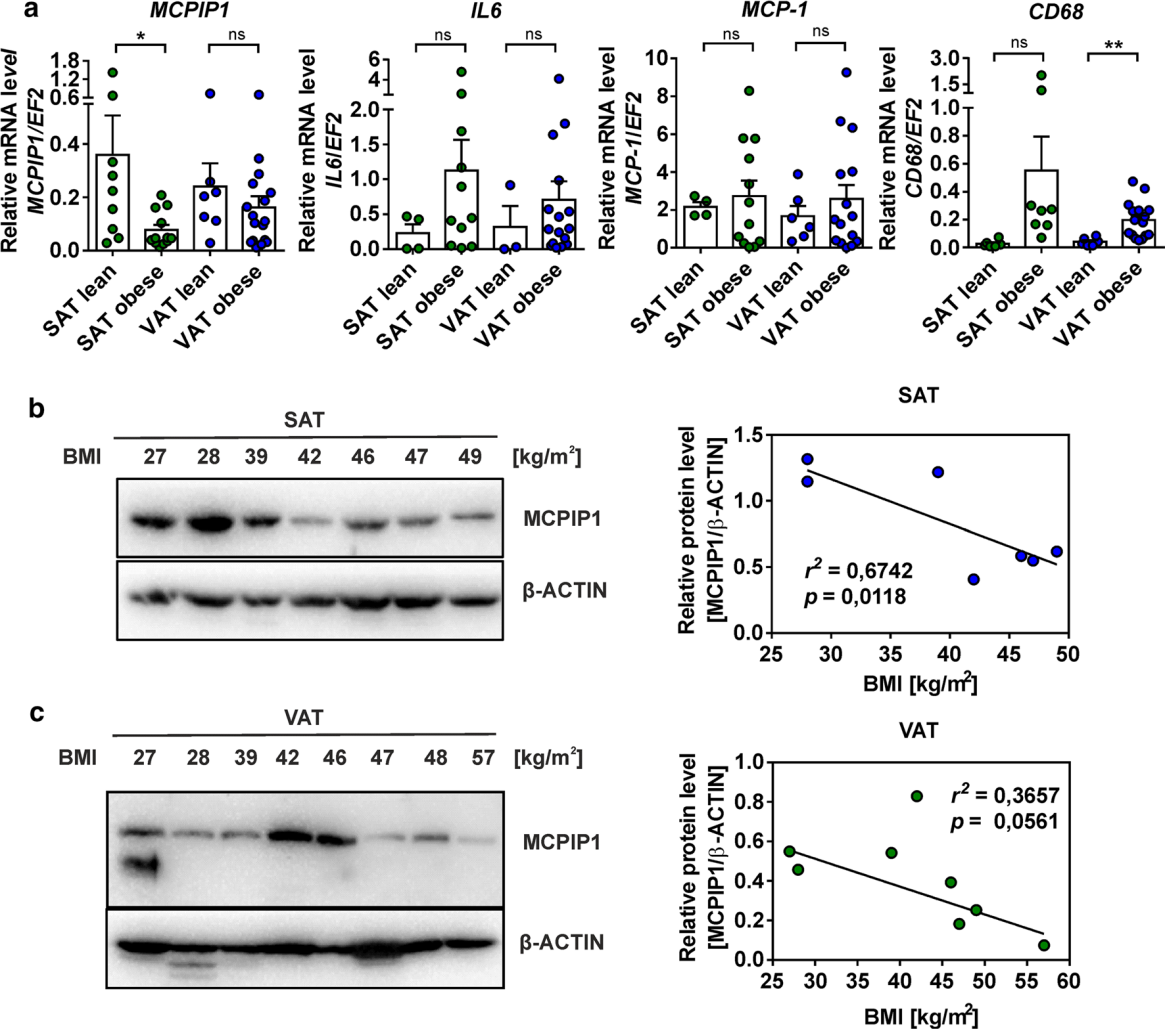
MCPIP1 levels in human adipose tissue of lean and obese subjects. **a** Scatter dot plot graph (mean ± SEM) showing expression analysis of MCPIP1, IL-6, MCP-1 and CD68 mRNA in SAT and VAT adipose tissues of lean (BMI < 28; *MCPIP1*: *n* = 9 for SAT and *n* = 7 for VAT; *IL6*: *n* = 4 for SAT and *n* = 3 for VAT; *MCP-1*: *n* = 4 for SAT and *n* = 6 for VAT; *CD68*: *n* = 6 for SAT and *n* = 6 for VAT) and obese (BMI > 30; *MCPIP1*: *n* = 12 for SAT and *n* = 17 for VAT; *IL6*: *n* = 11 for SAT and *n* = 16 for VAT; *MCP-1*: *n* = 12 for SAT and *n* = 15 for VAT; *CD68*: *n* = 8 for SAT and *n* = 16 for VAT) subjects estimated by Q-RT-PCR. Transcript levels were normalized to *EF2* mRNA levels. P values were estimated using two-tailed unpaired Student *t* test. **b** Left panel: Western blot analysis of MCPIP1 protein levels in subjects with different range of BMI in SAT (BMI: 27–49; *n* = 7) and **c** VAT (BMI: 27–57; *n* = 8). MCPIP1 protein levels were normalized to β-actin as the loading control. **b**, **c** Right panel presents the quantification of correlation between the MCPIP1 protein level and BMI in SAT and VAT. Statistical significance was determined with Pearson’s correlation. *p < 0.05; **p < 0.01

We analyzed MCPIP1 levels in biopsies of subcutaneous (SAT) and visceral (VAT) adipose tissue of lean and obese subjects. The group of obese patients was heterogenous and included individuals with type 2 diabetes. Our comparative analysis done for two studied reference control genes *EF2* and *ACTB*, has revealed lower deviations in Ct values for *EF2* in analyzed samples in comparison to Ct values for *ACTB* (Supplementary data, Fig. 1a), thus *EF2* was used as an internal control for the estimation of mRNA levels in adipose tissue biopsies. We observed statistically significant increase in the MCPIP1 transcript levels in SAT of lean subjects in comparison to obese subjects. In case of VAT the differences were not statistically significant (Fig. 1a). Furthermore, we observed a trend of higher levels of proinflammatory cytokines (*IL6* and *MCP-1*) in SAT and VAT of obese patients, however these differences were not statistically significant (Fig. 1a). VAT of obese patients was also characterized by an increased mRNA levels for CD68 being a macrophage marker. In the case of SAT, we observed a similar trend but the result was not statistically significant. Interestingly, we observed a correlation between MCPIP1 protein level and body mass index (BMI) in SAT (Fig. 1b), with a decreased levels of MCPIP1 protein along with an increased BMI (*r*^2^ = 0.67; *p* = 0.01; Fig. 1b). In VAT the correlation was not significant (*r*^2^ = 0.36; *p* > 0.05; Fig. 1c).

### Global transcriptome changes in 3T3-L1 adipocytes expressing wild-type MCPIP1

**Fig. 2:**
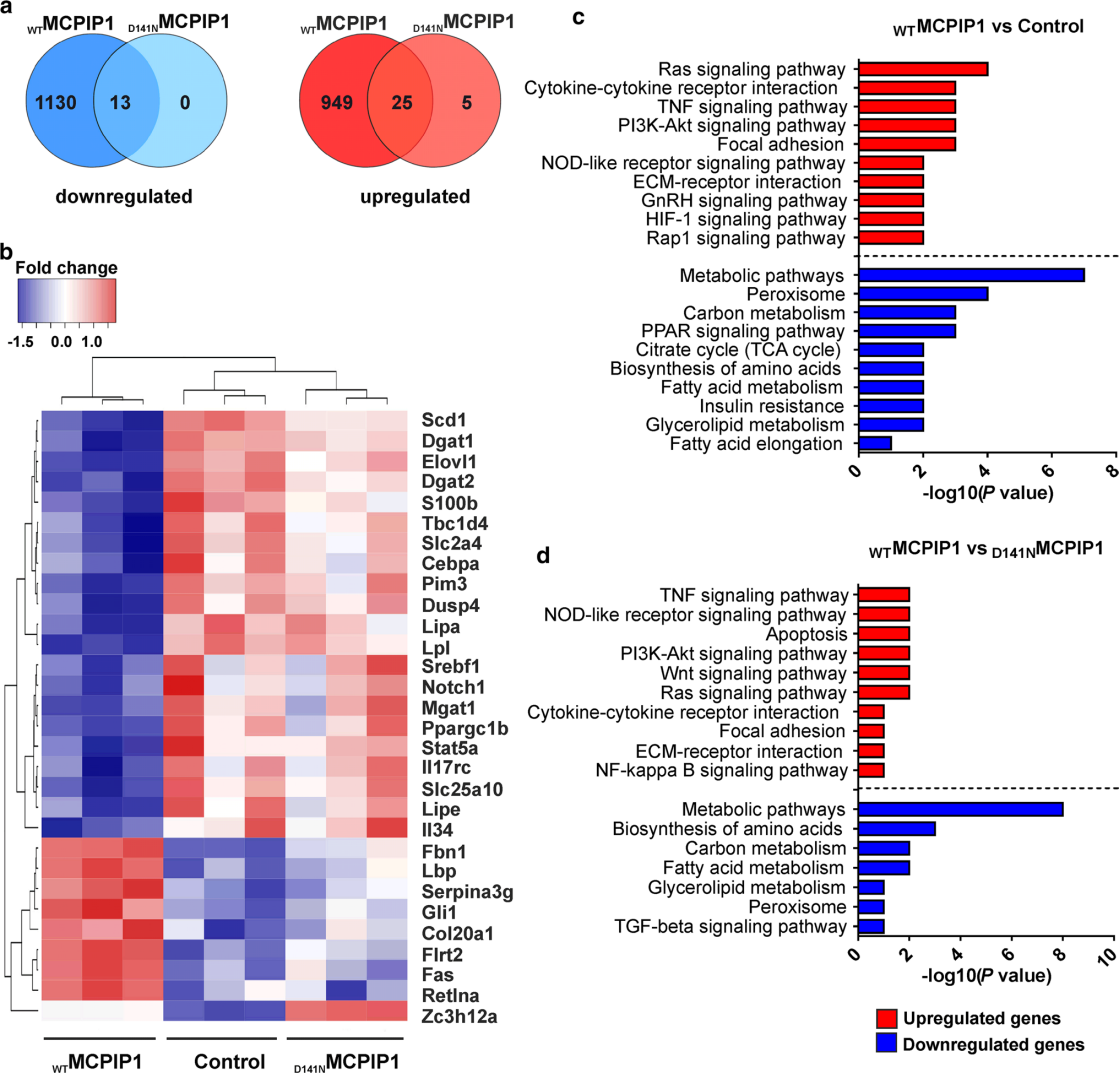
Global transcriptome analysis of 3T3-L1 adipocytes overexpressing wild-type or mutant MCPIP1 at day 2 of differentiation. **a** Venn diagrams present the number of differentially expressed transcripts (adj. *p* value < 0.05) in adipocytes expressing _WT_MCPIP1 and _D141N_MCPIP1 relative to the control cells (_WT_MCPIP1 vs. Control and _D141N_MCPIP1 vs. Control datasets; data was obtained for 3 biological replicates). **b** Heatmap of 30 genes that are associated with adipocyte biology and adipogenesis (adj. *p* value < 0.05; fold change ≥ 1.5). Each column represents an individual sample. **c** KEGG analysis of differentially expressed genes in _WT_MCPIP1 vs. Control and **d**_WT_MCPIP1 vs. _D141N_MCPIP1 adipocytes. Scale is the −log10 (*p* value) of the enrichment score. Red bars indicate upregulated genes and blue bars downregulated genes.

To assess the impact of MCPIP1 on the transcriptomic expression profile during adipogenesis, we performed RNA-Seq analysis of 3T3-L1 adipocytes at day 2 of differentiation, expressing wild-type (_WT_MCPIP1) or mutant MCPIP1 (_D141N_MCPIP1) in retroviral systems for three biological replicates. Adipocytes transduced with an empty vector (Control) were served as an experimental control. Relative gene expression levels were analyzed using gene expression ratios presented as log2 values. We identified 899 transcripts downregulated and 857 upregulated in _WT_MCPIP1 adipocytes in comparison to control cells (fold change ≥ 1.5; adj. *p* value < 0.05; data not shown). To establish transcripts for which expression might be dependent on RNase activity of MCPIP1, we compared gene expression levels in adipocytes expressing _WT_MCPIP1 and _D141N_MCPIP1. In this comparison, we identified 545 transcripts downregulated and 397 upregulated in _WT_MCPIP1 adipocytes (fold change ≥ 1.5; adj. *p* value < 0.05; data not shown). Next, we compared number of the differentially expressed genes in _WT_MCPIP1 and _D141N_MCPIP1 samples against control samples using pairwise comparison (adj. *p* value < 0.05). Analysis showed that 949 transcripts were upregulated and 1130 were downregulated in _WT_MCPIP1, whereas in _D141N_MCPIP1 samples, 5 transcripts were upregulated and none of them were detected as downregulated (Fig. 2a). Several differentially expressed transcripts were clustered by a visual heat map (Fig. 2b). Next, we performed functional annotation and classification of transcripts for which expression was altered in _WT_MCPIP1 in comparison to _D141N_MCPIP1 and control cells (groups _WT_MCPIP1 vs. Control and _WT_MCPIP1 vs. _D141N_MCPIP1; fold change ≥ 1.5; adj. *p* value < 0.05) using the DAVID bioinformatics resources (https://david.abcc.ncifcrf.gov/). The 707 downregulated transcripts were enriched in GO term belonging to 127 biological process (BP) in _WT_MCPIP1 vs. Control group (adj. *p* value < 0.05) and 404 transcripts to 107 BP in _WT_MCPIP1 vs. _D141N_MCPIP1 group (adj. *p* value < 0.05) (data not shown). The 781 upregulated transcripts in the _WT_MCPIP1 vs. Control group were enriched in 223 BP, whereas the 355 upregulated transcripts in the _WT_MCPIP1 vs. _D141N_MCPIP1 group were enriched in 107 BP (data not shown).

For further analysis we focused on molecular pathways that are mainly involved in the adipocyte differentiation process. Interestingly, numerous GO terms were common in both groups (_WT_MCPIP1 vs. Control and _WT_MCPIP1 vs. _D141N_MCPIP1; Supplementary data, Fig. 2a, b). The enlarged number of downregulated transcripts were involved mainly in metabolic, lipid metabolic and fatty acid metabolic process, whereas upregulated genes encoded proteins important for cell adhesion, multicellular organism development and negative regulation of peptidase activity (Supplementary data, Fig. 2a, b).

To determine pathways that are modulated by MCPIP1, KEGG enrichment analysis was performed. This analysis revealed 20 downregulated and 27 upregulated pathways in _WT_MCPIP1 vs. Control group (adj. *p* value < 0.05; data not shown), whereas, 19 downregulated and 19 upregulated pathways were detected in _WT_MCPIP1 vs. _D141N_MCPIP1 group (adj. *p* value < 0.05; data not shown). Similarly, as it was observed for GO terms, almost all of the enriched KEGG Pathways were common in both groups. Analogous to biological pathways, for the further analysis we chose only signaling pathways which are involved in adipogenesis (Fig. 2c, d). The top downregulated KEGG pathways include metabolic pathways, carbon metabolism and functioning of peroxisomes, while the Ras, TNF, and PI3K-Akt signaling pathways were the most upregulated in both groups (Fig. 2c, d).

### MCPIP1 regulates transcripts of factors involved in lipid metabolism

**Fig. 3:**
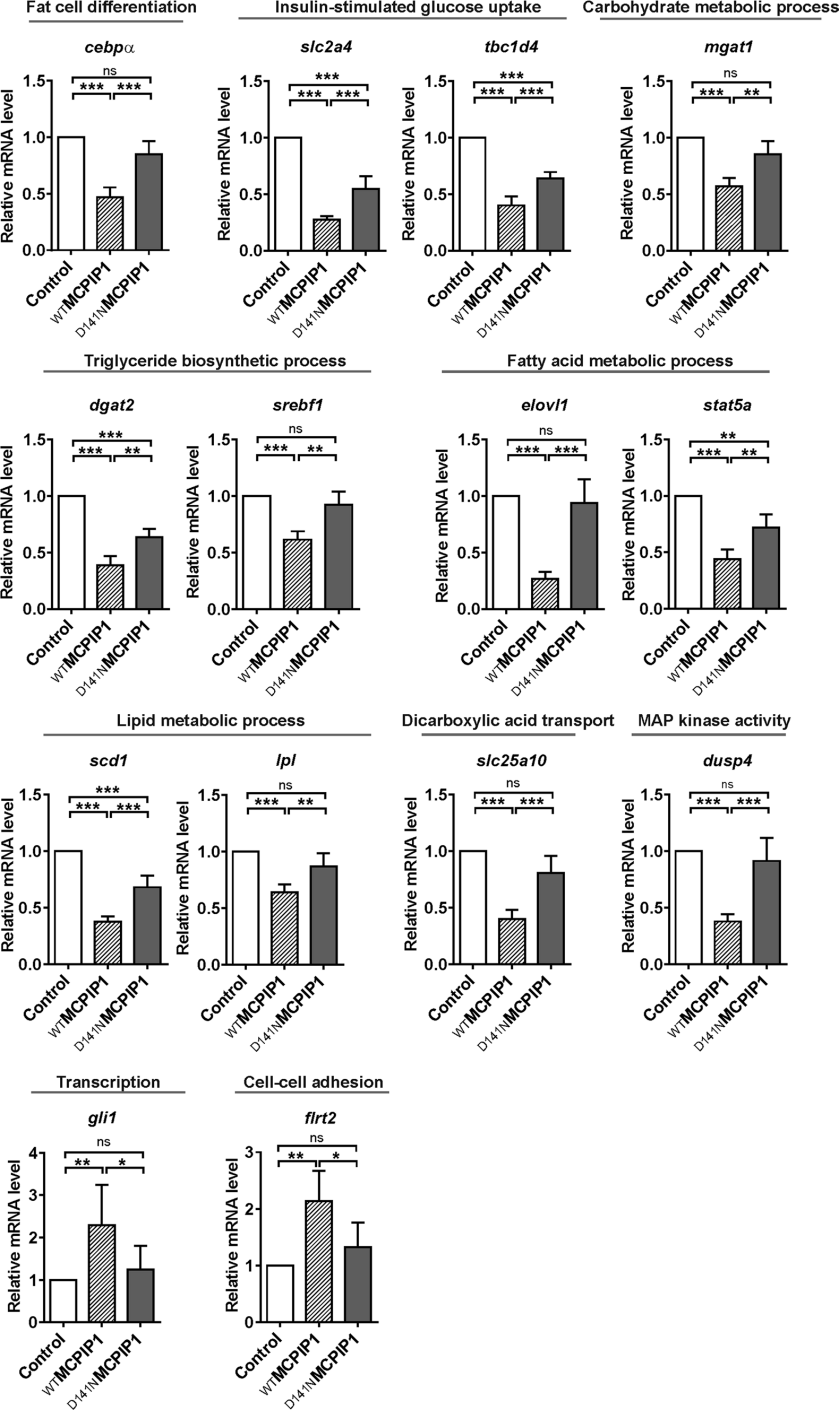
MCPIP1 decreases transcripts coding for proteins involved in lipid metabolism. Q-RT-PCR analysis of *cebpa*, *slc2a4*, *tbc1d4*, *mgat1*, *dgat2*, *srebf1*, *elovl1*, *stat5a*, *scd1*, *lpl*, *slc25a10*, *dusp4*, *gli1* and *flrt2* in Control, _WT_MCPIP1 and _D141N_MCPIP1 adipocytes at day 2 of differentiation. Transcript levels are normalized to *ef2* mRNA levels as a reference control. Data are presented as fold change (normalized to control cells) and as mean ± SD of four independent experiments. Statistical significance was determined with one-way ANOVA. *p < 0.05; **p < 0.01; ***p < 0.001

To verify the transcriptome data, we performed Q-RT-PCR using RNA isolated on the second day of differentiation from adipocytes transduced with retroviral vectors coding _WT_MCPIP1 or _D141N_MCPIP1 sequence or an empty vector, serving as an experimental control. Based on RNA-Seq analysis, we focused on transcripts that were downregulated in MCPIP1 overexpressing cells as possible targets of MCPIP1. Among these were genes known to be involved in fat cell differentiation (*cebpa*), insulin-stimulated glucose uptake (*slc2a4* and *tbc1d4*), carbohydrate metabolism process (*mgat1*), regulation of triglyceride biosynthesis process (*dgat2*, *srebf1*), fatty acid metabolic process (*elovl1*, *stat5a*), lipid metabolic process (*scd1*, *lpl*), dicarboxylic acid transport (*slc25a10*) and MAPK kinase activity (*dusp4*) (Fig. 3). In our studies we also checked transcripts with increased levels in _WT_MCPIP1 expressing adipocytes as compared to _D141N_MCPIP1 and control cells. One of these was involved in regulation of transcription (*gli1*) and the other one in cell–cell adhesion (*flrt2*) (Fig. 3). Q-RT-PCR analysis confirmed altered expression of selected transcripts between adipocytes expressing _WT_MCPIP1 and _D141N_MCPIP1 or Control cells and showed a high correlation with RNA-seq data (*p* < 0.0001, *r*^2^ = 0.9638, and *p* < 0.0001, *r*^2^ = 0.9056, between _WT_MCPIP1 vs Control and _WT_MCPIP1 vs _D141N_MCPIP1, respectively; Supplementary data Fig. 3a, b.

### Global proteome changes in 3T3-L1 adipocytes expressing wild-type or mutant MCPIP1

To better understand the mechanism underlying negative regulation of adipogenesis by MCPIP1, we assessed the proteomic profile of adipocytes using label-free mass spectrometry (LC–MS/MS) method. For this analysis we used 3T3-L1 adipocytes at day 4 of differentiation, stably transduced with retroviral vectors encoding wild-type MCPIP1 (_WT_MCPIP1) or mutant MCPIP1 (_D141N_MCPIP1) and an empty vector control.

**Fig. 4:**
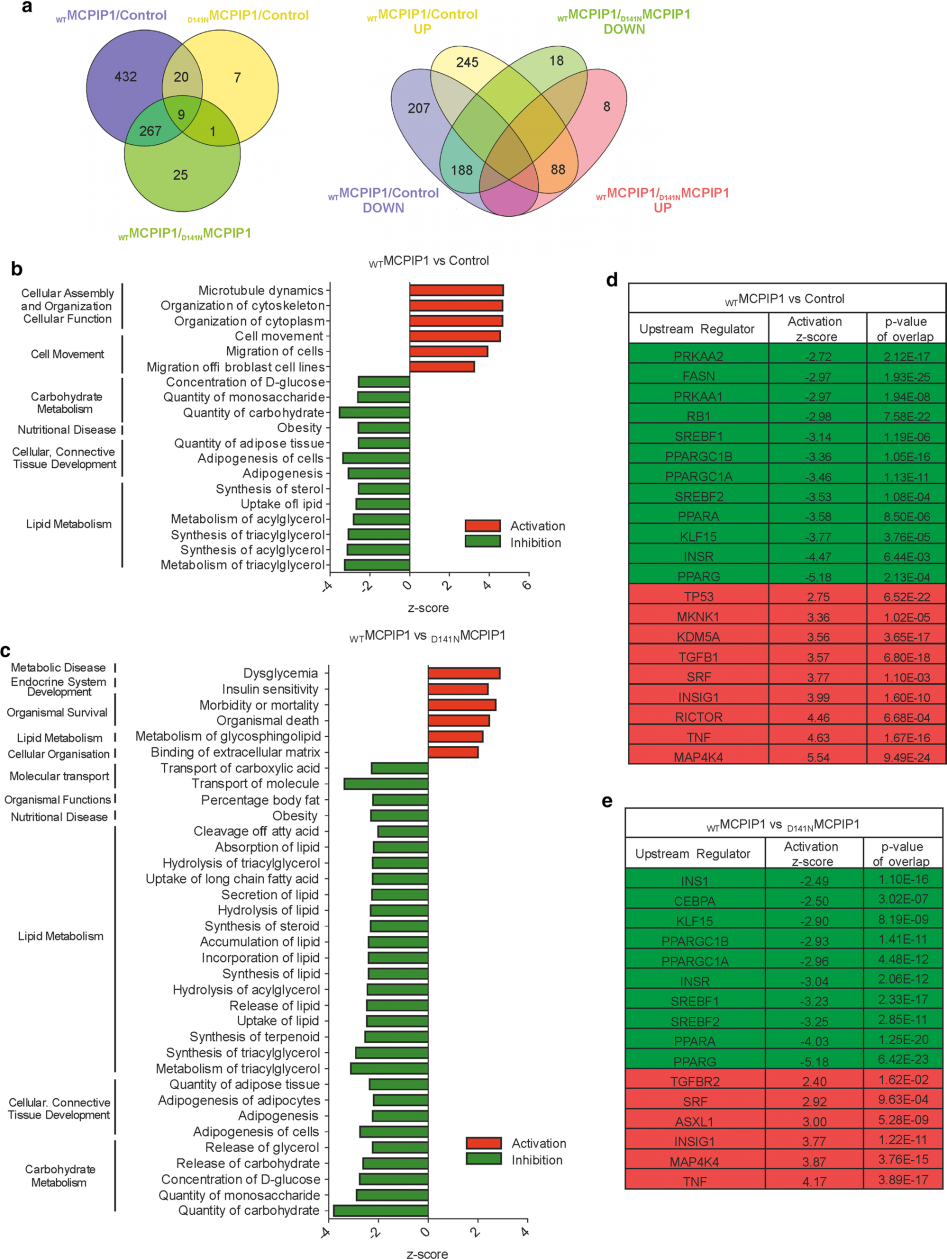
Global proteome changes in 3T3-L1 adipocytes overexpressing wild-type or mutant MCPIP1 at day 4 of differentiation. **a** Venn diagram comparison of proteomes (adj. *p* value < 0.05) from adipocytes expressing _WT_MCPIP1 or _D141N_MCPIP1 and control cells (left panel: _WT_MCPIP1 vs. Control; _WT_MCPIP1 vs. _D141N_MCPIP1 and _D141N_MCPIP1 vs. Control datasets; right panel: _WT_MCPIP1 vs. Control and _WT_MCPIP1 vs. _D141N_MCPIP1, both upregulated and downregulated proteins datasets; data were obtained for 3 biological replicates). **b** IPA analysis of significantly activated and inhibited biological processes in _WT_MCPIP1 vs Control and **c**_WT_MCPIP1 and _D141N_MCPIP1 group. Red and green bars indicate increased (*z*-score > 1) and decreased (*z*-score <  − 1) activation of biological process, respectively. **d** Potential upstream regulators predicted by IPA analysis (*p* value < 0.05) in _WT_MCPIP1 vs Control and **e**_WT_MCPIP1 and _D141N_MCPIP1 group. Proteins are indicated by their gene names. Red (*z*-score > 1) and green (*z*-score <  − 1) colors indicate increased and decreased protein levels, respectively.

LC–MS/MS analysis resulted in the identification of 728 proteins (395 downregulated and 333 upregulated) in _WT_MCPIP1 vs Control group, 302 proteins (206 downregulated and 96 upregulated) in _WT_MCPIP1 vs _D141N_MCPIP1 group, and 37 proteins (16 downregulated and 21 upregulated) in _D141N_MCPIP1 vs Control group (Fig. 4a).

To evaluate the molecular functions of the altered proteins in the three compared cell lines, we categorized them into biological processes, focusing on potential functions or diseases using a manually curated database and algorithms implemented in the IPA tool. A *z*-score was calculated to predict the activation status of analyzed biological processes or pathways. We identified 40 biological processes to be significantly altered in _WT_MCPIP1 adipocytes in comparison to control cells (data not shown). Furthermore, 41 processes were modulated with significant differences between the _WT_MCPIP1 and _D141N_MCPIP1 groups (data not shown). For the further analysis we focused on processes associated with adipocyte differentiation. Among potentially inhibited biological processes, the highest enrichment identified was for carbohydrate and lipid metabolism, in both _WT_MCPIP1 vs Control and _WT_MCPIP1 vs _D141N_MCPIP1 group (Fig. 4b, c). In turn, cellular organization and metabolic diseases showed the greatest enrichment among activated biological processes in _WT_MCPIP1 vs Control and _WT_MCPIP1 vs _D141N_MCPIP1 group, respectively (Fig. 4b, c).

To gain insight into the signaling pathways of the altered proteins, we matched and classified them into biological pathways provided by the Kyoto Encyclopedia of Genes and Genomes (KEGG) using the DAVID bioinformatics tool. We identified 40 and 28 pathways to be significantly downregulated and 34 and 7 pathways to be significantly upregulated in _WT_MCPIP1 vs Control and _WT_MCPIP1 vs _D141N_MCPIP1 group, respectively (data not shown). Similar to biological process analysis, in the further studies we selected pathways, which are associated with the regulation adipocyte biology. The most inhibited pathways for the altered proteins in both _WT_MCPIP1 vs Control and _WT_MCPIP1 vs _D141N_MCPIP1 groups were metabolic pathways, followed by carbon and fatty acid metabolism (Supplementary Fig. 4a, b). The most activated pathway in both groups was lysosome function. Additionally, in _WT_MCPIP1 vs Control comparison, we also identified enrichment for pathways involved in regulation of actin cytoskeleton and focal adhesion (Supplementary Fig. 4a).

We also conducted functional network analysis using IPA and found potential upstream regulators of both groups. PPARγ was predicted to be the most inhibited in _WT_MCPIP1 as compared to Control and _D141N_MCPIP1 cells (Fig. 4d, 4e). Conversely, the most activated potential upstream regulators were MAP4K4 in the _WT_MCPIP1 vs Control group was MAP4K4 (Fig. 4d) and TNF in _WT_MCPIP1 vs _D141N_MCPIP1 group (Fig. 4e).

### Comparison of transcriptomics and proteomics data in 3T3-L1 adipocytes expressing wild-type or mutant MCPIP1

To compare the concordance of transcriptome and proteome changes in a given comparison we calculated Pearson's correlation using fold changes as an input. All correlation coefficients were significant (*p* value < 0.05) and reached 0.31, 0.34 and − 0.23 for _WT_MCPIP1 vs Control, _D141N_MCPIP1 vs Control and _WT_MCPIP1 vs _D141N_MCPIP1 comparison, respectively. Since the coefficients values suggested moderate association between RNA-seq and MS dataset on expression level in the next step we compared these two datasets functionally using Gene Ontology and KEGG Pathways annotations. This comparison identified multiple pathways that are crucial for adipocyte differentiation.

To compare common biological processes assessed for transcriptome and proteome we used predicted GO terms for the differentially expressed genes, taking into account their activation status (calculated *z*-score), and evaluated the proteome data using the same IPA tool. The list of processes in common between the transcriptome and proteome data is presented in the supplementary data (Supplementary Fig. 5a, b). Of note, we found that overlapped downregulated/inhibited processes were mainly involved in carbohydrate metabolism, lipid metabolism and cellular development in both _WT_MCPIP1 vs Control and _WT_MCPIP1 vs _D141N_MCPIP1 groups (Supplementary Fig. 5a, b). In turn, common upregulated/activated processes included cellular assembly and cell movement for _WT_MCPIP1 vs Control group (Supplementary Fig. 5a), and organismal survival in the _WT_MCPIP1 vs _D141N_MCPIP1 group (Supplementary Fig. 5b).

The results of the KEGG signaling comparison showed 13 and 7 common pathways to be downregulated in both the transcriptome and proteome data for the _WT_MCPIP1 vs Control and _WT_MCPIP1 vs _D141N_MCPIP1 groups, respectively (Supplementary Fig. 6a, b). For both groups, the highest enrichment observed was for metabolic pathways, followed by carbon metabolism and fatty acid metabolism (Supplementary Fig. 6a, b). In case of activated pathways, we identified 3 common pathways including the Ras signaling pathway, focal adhesion, and Rap1 signaling pathway for the _WT_MCPIP1 vs Control group (Supplementary Fig. 6c). However, we did not find overlapped pathways involved in adipogenesis in comparison of _WT_MCPIP1 adipocytes and _D141N_MCPIP1 cells.

The transcriptome and proteome datasets, although moderately correlated, have a high concordance for multiple processes and pathways involved in the control of adipocyte biology. The results clearly show that adipocytes overexpressing _WT_MCPIP1 are characterized by downregulation or inhibition of key processes and pathways necessary for adipocyte differentiation. Observed modulation of genes expression triggered by _WT_MCPIP1 is specific as profile of changes is characteristic only for cells expressing wild-type protein and not by for both controls: cells expressing mutant form of MCPIP1 and/or cells transduced with an empty vector.

### MCPIP1 impairs insulin-stimulated glucose uptake in adipocytes

**Fig. 5:**
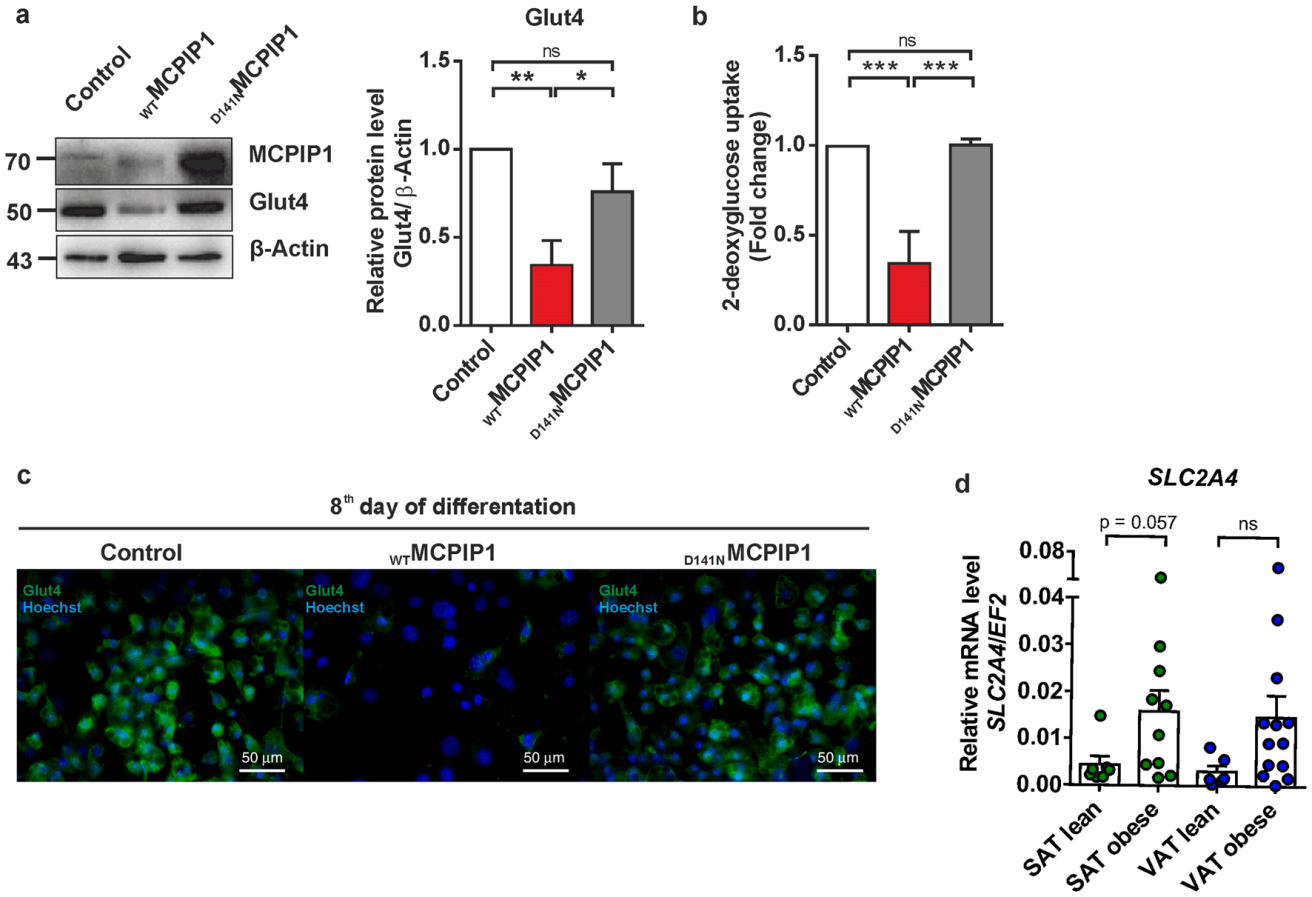
MCPIP1 impairs glucose uptake in 3T3-L1 adipocytes **a** Western blot analysis of MCPIP1, Glut4 with β-actin as the loading control in 3T3-L1 adipocytes overexpressing wild-type or mutant MCPIP1 and control cells at day 8 of differentiation. Left panel: representative Western blot. Right panel: densitometric quantification of Glut4 protein normalized to β-Actin. Data are presented as fold change (normalized to control cells) and as mean ± SD of three independent experiments. **b** Analysis of insulin-stimulated 2-deoxyglucose (2-DG) uptake in 3T3-L1 adipocytes overexpressing wild-type or mutant MCPIP1 and control cells at day 8 of differentiation. Data are presented as fold change (normalized to control cells) and as mean ± SD of three independent experiments. Statistical significance was determined with one-way ANOVA. *p < 0.05; **p < 0.01; ***p < 0.001 **c** Immunofluorescent staining for Glut4. Representative merged images of 3T3-L1 adipocytes overexpressing wild-type or mutant MCPIP1 and control cells at day 8 of differentiation (Hoechst 33,258 dye used for nuclei; Glut4 antibody labeled with fluorescent dye AlexaFluor 488). Scale bar represents 50 μm. **d** Scatter dot plot graph (mean ± SEM) showing expression analysis of SLC2A4 mRNA in SAT and VAT adipose tissues of lean (BMI < 28; *n* = 7 for SAT and *n* = 6 for VAT) and obese (BMI > 30; *n* = 10 for SAT and *n* = 13 for VAT) subjects estimated by Q-RT-PCR. Transcript levels were normalized to *EF2* mRNA levels. *p* values were estimated using two-tailed unpaired Student *t* test

Our RNA-seq data showed a significant decrease in expression of genes encoding proteins responsible for insulin-stimulated glucose uptake (i.e., *slc2a4* and *tbc1d4*, Figs. 2b, 3) in _WT_MCPIP1 adipocytes as compared to _D141N_MCPIP1 and control cells. This led us to investigate whether MCPIP1 can modulate glucose uptake in 3T3-L1 adipocytes. First, we measured protein levels of the glucose transporter, Glut4 (encoded by *slc2a4* gene) in _WT_MCPIP1, _D141N_MCPIP1, or control 3T3-L1 adipocytes at day 8 of differentiation. We observed a significant decrease in protein level of Glut4 within _WT_MCPIP1 adipocytes as compared to _D141N_MCPIP1 and control cells (Fig. 5a) and confirmed this results using immunofluorescence (Fig. 5c). In addition, we assessed the effects of MCPIP1 overexpression on glucose uptake. As shown in Fig. 5b 2-deoxyglucose (2-DG) uptake was significantly reduced in _WT_MCPIP1 adipocytes as compared to mutant and control cells. We also observed a trend of the higher level of GLUT4 mRNA (*SLC2A4*), although this difference was not statistically significant between lean and obese patients, however these results exhibited negative correlation with MCPIP1 mRNA levels (Figs. 1a, 5d).

Glut4 is one of the adipogenic factors essential for adipocyte differentiation, which expression is driven by key transcription factors, including PPARγ and C/EBPα. We examined, whether decreased Glut4 level observed in adipocyte overexpressing _WT_MCPIP1 might be caused by downregulation of PPARγ and C/EBPα. For this purpose, we used Western blot and evaluated the protein profile of these transcription factors in adipocytes overexpressing _WT_MCPIP1, _D141N_MCPIP1 and control cells at the different time points during adipogenesis. The data showed significantly decreased levels of PPARγ and C/EBPα at day 4 and 8 of differentiation in adipocytes overexpressing _WT_MCPIP1 adipocytes in comparison to adipocytes overexpressing _D141N_MCPIP1 (Supplementary Fig. 7a). Moreover, these changes were correlated with the diminished ability to accumulate lipid droplets by _WT_MCPIP1 adipocytes, assessed by the Oil-red staining (Supplementary Fig. 7b).

### Enforced expression of Glut4 rescues adipogenesis in 3T3-L1 adipocytes expressing wild-type MCPIP1

**Fig. 6:**
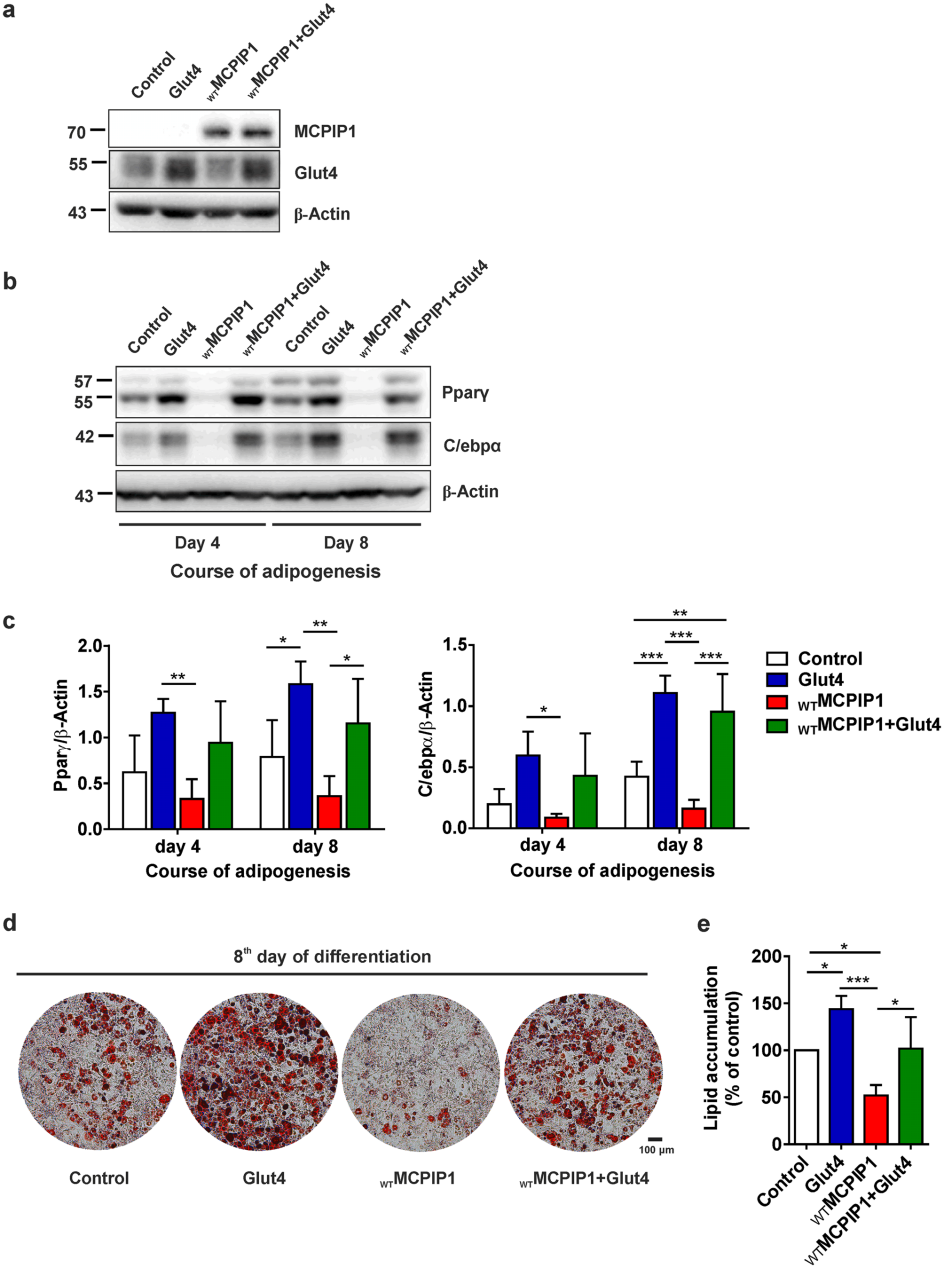
Enforced expression of Glut4 rescues differentiation in 3T3-L1 adipocytes expressing wild-type MCPIP1. **a** Representative Western blot for MCPIP1 and Glut4 protein levels from total cell lysates of _WT_MCPIP1, Glut4, _WT_MCPIP1 + Glut4 and adipocytes with an empty vector (Control) at day 0 of differentiation. **b** Representative Western blot for PPARγ and C/EBPα from total cell lysates of _WT_MCPIP1, Glut4, _WT_MCPIP1 + Glut4 and adipocytes with an empty vector (Control) at day 4 and 8 of differentiation. **c** Densitometric quantification of PPARγ (*n* = 4) and **d** C/EBPα (*n* = 4) protein levels normalized to β-Actin and presented as mean ± SD. Statistical significance was determined with one-way ANOVA. *p < 0.05; **p < 0.01; **p < 0.001 **e** Oil O Red staining of lipid droplets accumulated by 3T3-L1 adipocytes overexpressed wild-type MCPIP1, Glut4 or both wild-type MCPIP1 and Glut4 and control cells at day 8 of adipogenic differentiation. The pictures represent data from one of four independent experiments. Scale bar represents 100 μm. **f** Graph presenting percentage of accumulated lipid droplets estimated by measuring the absorbance of stain extracted with 100% isopropanol. Data are presented as mean ± SD of four independent experiments (normalized to control cells). Statistical significance was determined with one-way ANOVA. *p < 0.05; ***p < 0.001

Our data reveal that MCPIP1 impairs adipogenesis by decreasing Glut4 level. Thus, we were interested, if this effect could be reversed by retroviral expression of Glut4 in _WT_MCPIP1-overexpressed 3T3-L1 cells. For this purpose, we generated 3T3-L1 cells with stable expression of Glut4 alone or MCPIP1 together with Glut4. In the latter case, 3T3-L1 cells infected previously with retroviruses expressing wild-type MCPIP1, were infected again with vectors coding for Glut4 mRNA. To check whether enhanced Glut4 in adipocytes expressed _WT_MCPIP1 will rescue negative effect of MCPIP1 on adipogenesis, 3T3-L1 preadipocytes with _WT_MCPIP1, Glut4 or _WT_MCPIP1 together with Glut4 expression and control cells, with an empty vector, were induced to adipocyte differentiation. First, we confirmed enforced expression of either MCPIP1, Glut4 or both MCPIP1 and Glut4 in cell lysates, harvested at the day of differentiation induction (day 0) by Western blot using antibodies against MCPIP1 and Glut4 (Fig. 6a). Ectopic expression of Glut4 rescued adipogenesis in _WT_MCPIP1-overexpressing 3T3-L1 adipocytes, as corroborated by significantly increased protein levels of adipogenesis markers PPARγ (Fig. 6b, c) and C/EBPα (Fig. 6b, d) at day 8 of differentiation in comparison to adipocytes expressed _WT_MCPIP1 alone. In addition, lipid accumulation, evaluated by ORO staining were comparable between control and adipocytes co-transduced with MCPIP1 and Glut4 (Fig. 6e, f). In contrast, for 3T3-L1 adipocytes expressed Glut4 we observed enhanced adipogenesis, as defined by increased protein level of PPARγ (Fig. 6b, c) and C/EBPα (Fig. 6b, d) at day 4 and 8 of differentiation and augmented lipid accumulation (Fig. 6e, f) in comparison to control cells. From these data we suggest, that the decreased level of Glut4 observed in 3T3-L1 adipocytes expressing _WT_MCPIP1 leads to the reduced adipogenesis.

### MCPIP1 diminishes insulin receptor activation and downstream signaling

**Fig. 7:**
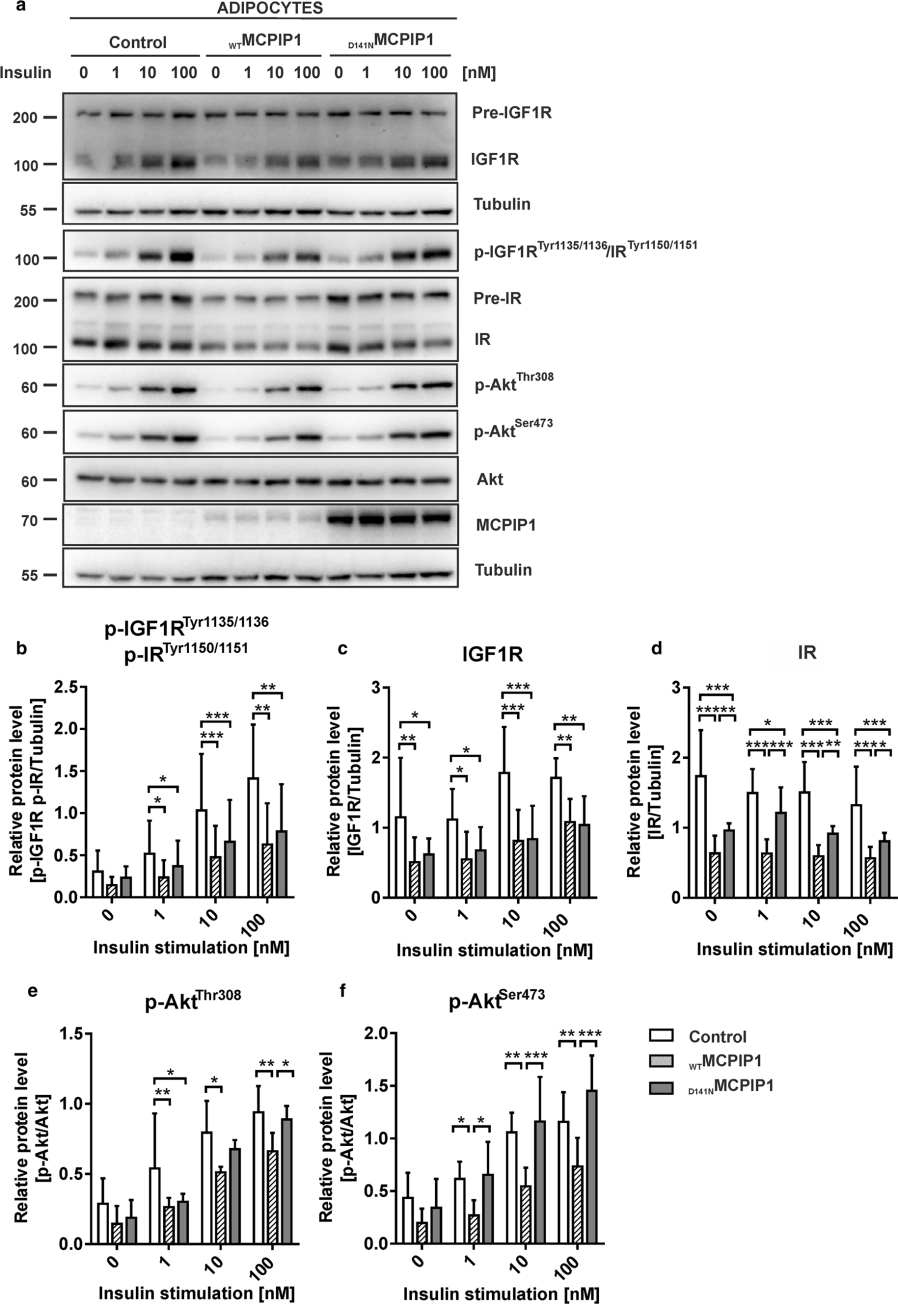
MCPIP1 diminishes insulin receptor activation and downstream signaling in 3T3-L1 adipocytes. **a** Representative Western blots from total cell lysates of _WT_MCPIP1, _D141N_MCPIP1 and adipocytes with an empty vector (Control) at day 8 of differentiation without (0 nM) and after 15 min of 1, 10 or 100 nM insulin stimulation. **b** Quantification of tyrosine phosphorylated IGF1R/IR in 3T3-L1 adipocytes, after insulin stimulation, normalized to tubulin level (*n* = 3). **c** Quantification of total IGF1R protein level, after insulin stimulation in 3T3-L1 adipocytes, normalized to tubulin level (*n* = 3). **d** Quantification of total IR protein level, after insulin stimulation in 3T3-L1 adipocytes, normalized to tubulin level (*n* = 4). **e** Quantification of p-Akt Thr308 and **f** p-Akt Ser473 protein level, after insulin stimulation in 3T3-L1 adipocytes, normalized to total Akt level (*n* = 4). Data are presented as mean ± SD of three or four independent experiments (as indicated above). Statistical significance was determined with two-way ANOVA. *p < 0.05; **p < 0.01; ***p < 0.001

Based on our proteome and functional network analysis that revealed insulin receptor as one of the most strongly inhibited upstream regulator in _WT_MCPIP1 expressing cells comparing to the Control and _D141N_MCPIP1 expressing cells (Fig. 4d, e) and the fact, that _WT_MCPIP1 attenuates insulin-stimulated glucose uptake in adipocytes, we decided to check whether MCPIP1 might modulate insulin signaling pathway. To further characterize the effects of MCPIP1 on insulin sensitivity, we examined changes in the activities of the IGF1R/IR and downstream signaling proteins, including Akt. To investigate effects of MCPIP1 on IR activation, adipocytes stably transduced with retroviral vectors encoding _WT_MCPIP1 or _D141N_MCPIP1, and an empty vector were serum-starved for 2 h at the day 8 of differentiation, followed by stimulation with insulin (0, 1, 10 and 100 nM) for 15 min (Fig. 7a).

Insulin stimulation of 3T3-L1 adipocytes revealed significant reduction in IGF-1 receptor (IGF1R) and IR phosphorylation (Tyr1135/1136 and Tyr1150/1151) in _WT_MCPIP1 and slightly diminished decrease in _D141N_MCPIP1 adipocytes in comparison to control cells (Fig. 7b). Moreover, the total level for IGF1R protein was also decreased in adipocytes overexpressing _WT_MCPIP1 and _D141N_MCPIP1 in comparison to control adipocytes (Fig. 7c). Interestingly, we found that _WT_MCPIP1 significantly reduced total protein level of IR in comparison to both _D141N_MCPIP1 and empty (Fig. 7d). The reduced IGF1R/IR activation as well as decreased total level of IR resulted in the reduction of Akt phosphorylation on both Thr308 and Ser473 positions in _WT_MCPIP1 adipocytes in comparison to _D141N_MCPIP1 and control cells (Fig. 7e, f). Furthermore, Ser473 phosphorylation declined more significantly than Thr308 in the _WT_MCPIP1 adipocytes (Fig. 7f). These results might explain reduced glucose uptake in _WT_MCPIP1 adipocytes via attenuated insulin signaling pathway in this cells.

## Discussion

Obesity is a rapidly increasing global health problem in developed countries, with an easy access to food and significantly reduced physical activity leading to excessive accumulation of adipose tissue in a large percentage of the population. One significant consequence of obesity is systemic, chronic inflammation characterized by infiltration of macrophages and increased release of bioactive substances, such as cytokines/chemokines and adipokines. Adipose tissue inflammation impairs organismal homeostasis and contributes to the development of obesity-related diseases [29–32]. However, there are two main compartments of adipose tissue, with different metabolic characteristics: subcutaneous adipose tissue (SAT) and visceral adipose tissue (VAT). VAT has multiple endocrine, metabolic, and immunological functions and is more strongly associated with obesity-related risk factors than SAT [29]. Moreover, VAT preadipocytes exhibit less differentiation potential and possess an increased proportion of large adipocytes that are more prone to develop insulin resistance in comparison with SAT adipocytes [30–33].

MCPIP1 was discovered as an anti-inflammatory agent. While its role in adipose tissue development is well described, the data are sometimes contradictory. Younce and collaborators showed that MCPIP has a strong differentiation potential and can induce adipogenesis without activity of PPARγ [34]. Similarly, Habacher et al. demonstrated that *C. elegans*, when adapted to stressful conditions caused by decreasing temperature, overexpresses REGE-1, a protein exhibiting close homology to MCPIP1 [35]. This protein controls body fat by targeting the 3′ UTR of an mRNA encoding a fat-loss-promoting transcription factor, ETS-4. In turn, we showed that MCPIP1 negatively regulates adipogenesis by degrading transcripts coding for C/EBPβ [13, 15]. Observations made by Li et al. were similar to ours, demonstrating that transcripts coding for C/EBPβ and C/EBPδ are direct targets of MCPIP1 RNase [36].

We carried out in vitro experiments using 3T3-L1 cells, and we analyzed MCPIP1 levels in SAT and VAT isolated from patients with different BMI. We observed higher levels of MCPIP1 transcript in tissue from lean patients than obese patients in SAT. Surprisingly, these data do not agree with our previously presented data showing that MCPIP1 transcript levels increases with BMI [13]. However, this phenomena might be explained by the type of control used in the previous experiment. In those studies fat tissue from women with breast cancer was used as a control. In contrast, our current study utilized control samples from lean patients having inguinal hernia. The levels of proinflammatory markers in fat tissue of lean patients (IL-6 and MCP-1) exhibited a trend to be lower than in obese patients and MCPIP1 transcript levels negatively correlated with transcripts coding for these markers in SAT. The fact that the differences are not statistically significant results probably from the use of tissues from a small and heterogeneous group of obese patients, as well as small control group. The levels of transcript coding for macrophage marker, CD68 was higher in VAT of obese when in case of SAT this difference was not statistically significant, however these data negatively correlate with the levels of MCPIP1 in SAT and VAT of lean and obese patients. Interestingly, we observed decreased MCPIP1 level in SAT along with increased BMI, including morbidly obese individuals, with BMI above 40 kg/m^2^, suggesting that MCPIP1 is negatively regulated in subcutaneous adipose tissue of obese patients. Furthermore, these findings are consistent with our in vivo studies where the murine model of diet-induced obesity was used. We observed decreased MCPIP1 level in adipose tissue of male C57BL/6 mice fed a high-fat diet for 12 weeks in comparison to animals fed a control diet [37]. Previous studies showed that macrophages exhibit the high levels of MCPIP1 expression [4]. Thus, correlation between low MCPIP1 levels and high CD68 levels in fat tissue of morbidly obese patients may indicate that MCPIP1 is downregulated in adipocytes and not in macrophages. Albeit, this hypothesis has to be clarified. Furthermore, it has to be clear out how MCPIP1 is downregulated in fat tissue of obese patients.

To further understand the role of MCPIP1 in adipose tissue homeostasis and the reason for its downregulation in obese individuals, we carried out transcriptome and proteome analyses in differentiating 3T3-L1 at the day 2 and 4, respectively. Transcriptomic analysis showed a significantly higher number of differentially expressed molecules than proteomic analysis. Upregulated transcripts encoded proteins involved in signal transmission, in particular Ras, TNF and Rap and PI3K–Akt, while downregulated transcripts encoded proteins important in metabolism, biosynthesis of amino acids and peroxisome activity. In case of proteome data we noticed that MCPIP1 expressing adipocytes exhibited upregulation of proteins involved in cellular organization and movement while downregulated proteins were associated with lipid and carbohydrate metabolism, and in particular, lipid uptake and accumulation, synthesis of steroids, release of lipids, glycerol and carbohydrates. Negative regulation was also observed for proteins involved in connective tissue development and adipogenesis.

In both transcriptome and proteome data sets we were able to determine differentially expressed molecules involved in the same biological processes. Downregulated transcripts/proteins were involved in carbohydrate and lipid metabolism, peroxisome activity and cellular development when upregulated molecules are crucial for cellular assembly and cell movement. Given the RNase activity of MCPIP1, negatively regulated transcripts/proteins could be direct downstream targets. However, these molecules may also be under the control of CEBPβ and PPARγ—two transcription factors that positively regulate adipocyte differentiation and are downregulated by MCPIP1 [13, 15]. Besides its role in adipocyte differentiation, PPARγ is also thought to play a role in lipid metabolism, and insulin action through coordinated effects on gene transcription [38].

Our global transcriptome analysis showed decreased expression of genes encoding proteins for which a role in the adipocyte development is well documented. Among these genes we found transcripts important for lipid metabolism including elongation of very-long-chain fatty acids 1 (*elovl1*). This protein was shown to enhance triacylglycerol synthesis and subsequent accumulation of lipid droplets in 3T3-L1 expressing cells [39]. MCPIP1 expressing adipocytes also exhibited downregulation of transcripts encoding stearoyl-CoA desaturase 1 (*scd1*), a protein responsible for lipid metabolism/global adipocyte lipid composition and upregulated during adipogenesis [40]. These data show, that MCPIP1 could positively impact adipocyte metabolism by decreasing accumulation of lipid droplets. However, impaired accumulation of lipids in fat tissue leads to their increased accumulation in the blood and surrounding tissues, such as liver and muscle, inducing pathological effects. Thus, reduced levels of MCPIP1 in adipose tissue of morbidly obese individuals may be beneficial for other tissues. Similarly, MCPIP1 decreases the levels of Glut4, a protein involved in insulin-stimulated glucose uptake (both on the mRNA and protein level). It was shown that age-related reduction of expression the Glut4 encoding gene, s*lc2a4*, is associated with the development of decreased glucose tolerance and insulin resistance [41, 42]. According to Dalen and coworkers, the gene coding for Glut4 is regulated by PPARγ activators in mature adipocytes [43]. Our results are in accordance with these observations as exogenous expression of MCPIP1 leads to downregulation of C/EBPα and PPARγ and as a consequence it might be one of the mechanisms of downregulation of the Glut4 transcript and protein. We suggest that 3T3-L1 cells with stable _WT_MCPIP1 expression showed impaired adipogenesis presumably due to decreased Glut4 level. We draw this conclusion because the differentiation impairment of _WT_MCPIP1-expressed 3T3-L1 adipocytes has been rescued by the overexpression of Glut4 in this cells.

Similarly, downregulation of the transcript encoding Tbc1d4, a protein (also known as AS160) important in the insulin-stimulated translocation of the glucose transporter Glut4 from intracellular vesicles to the plasma membrane, leads to increased adipocyte glucose uptake [44]. Moreover, for Glut4 mRNA level we observed a trend of increased expression in both SAT and VAT of obese subjects in comparison to lean group. The expression of SLC2A4 was furthermore inversely correlated with MCPIP1 expression in these tissues. Again, strong downregulation of MCPIP1 in adipose tissue of patients with high BMI may promote glucose uptake by adipocytes to protect liver and muscles.

These results led us to investigate whether MCPIP1 might modulate insulin sensitivity of adipocytes. Stimulation of adipocytes with different range of insulin revealed changes manifested by alterations of phosphorylation status of major factors involved in insulin signaling, including IGF1R/IR and Akt. A key action of insulin is to stimulate glucose uptake by activating the translocation of the glucose transporter (Glut4) from intracellular compartment to the plasma membrane. Insulin binds to IR or to IGF1R/IR heterodimer what results in the cascade of phosphorylations of the downstream signaling molecules [45]. One of the key factor required for insulin-stimulated glucose uptake is Akt [46]. Phosphorylation of Akt in Thr308 and Ser473 positions results in phosphorylation of AS160/TBC1D4, proteins responsible for glucose transport [44]. We showed that MCPIP1 overexpression in 3T3-L1 adipocytes significantly decreases the phosphorylation of IGF1R/IR in comparison to control cells and the effect is stronger for wild-type than mutated form of MCPIP1. Moreover, _WT_MCPIP1, but not _D141N_MCPIP1 expressing adipocytes were characterized by a significantly decreased basal level of IR. As a result, _WT_MCPIP1 expressing cells exhibited also decreased activation of Akt what in consequence leads to inhibition of insulin-glucose uptake and adipocyte differentiation. These observations can be referred to the results of Groeneveld and collaborators [47]. They analyzed IR expression during adipogenesis and showed that knockdown of this receptor after adipocytes’ differentiation severely attenuates insulin-induced Akt phosphorylation and 2-deoxyglucose uptake. Furthermore, reduction of IR expression after 14 days of differentiation triggers delipidation of the cells [47].

Besides the effect of MCPIP1 on insulin signaling and glucose uptake we observed its influence on the negative regulation of key transcription factors that promote adipogenesis including CCAAT/enhancer-binding protein alpha (*cebpa*), signal transducer and activator of transcription-5a (*stat5a*), and cyclic AMP response element-binding protein (*creb*). Among upregulated genes, we identified *gli1*, which encodes GLI Family Zinc Finger 1 (*gli*), member of the Hedgehog (Hh) protein family. Hh signaling plays a conserved role in inhibition of the adipogenic program [48].

Another interesting observation was decreased expression of several genes involved in peroxisome biogenesis and assembly. The major function of the peroxisome is the breakdown of very-long-chain fatty acids through beta-oxidation and reduction of reactive oxygen species [49, 50]. The importance of peroxisomes for adipocyte function is poorly understood. However, the number of peroxisomes was shown to increase during differentiation of adipogenic cell lines [51]. Moreover, a reduction in the number of peroxisomes results in peroxisomal fatty acid oxidation causing accumulation of long- and very long-chain (polyunsaturated) fatty acids [52]. Interestingly, peroxisomes were also found to be involved in lipid biosynthesis [51]. In light of our data, we suggest that impaired lipid metabolism in MCPIP1-overexpresing adipocytes might be also the consequence of decreased peroxisome activity.

The integrative transcriptome and proteome analyses presented here show that in addition to the well-established anti-inflammatory role of MCPIP1, this protein is also an important regulator of adipogenesis and adipocytes metabolism. Our data partially address this key question, why morbidly obese patients have low MCPIP1 levels in fat tissue. Based on our observation we can assume that decreased MCPIP1 levels force adipocytes to accumulate lipids and enhance glucose uptake. This means that the fat tissue environment in obese generates conditions favorable to hypertrophy to increase the adipocyte’s ability to accumulate lipids. However, most studies support a reduction of adipogenesis in the obese state. Probably, elevated levels of proinflammatory cytokines in adipose tissue of obese subjects, which might be result of lowered MCPIP1 levels in this tissue, affect the rate of hyperplasia. Moreover, it has been shown that MCPIP1 is mainly involved in the acute phase of inflammation, while in the chronic phase, presented in obese conditions, other proteins may take the anti-inflammatory control (e.g., Roquin) [1]. Thus, the interplay of MCPIP1, cytokines and lipid metabolism needs more studies. Additionally, further studies are interesting in terms of searching for MCPIP1 inhibitors in fat tissue of obese subjects and answer the question why MCPIP1 is inhibited.

## Electronic supplementary material

Below is the link to the electronic supplementary material.

Supplementary file1 (DOCX 13 kb)

Supplementary file2 (XLSX 259 kb)

Supplementary file3 (DOCX 16 kb)

Supplementary Fig. 1 Evaluation of internal control gene level for mRNA expression in human adipose tissue of lean and obese subjects. **a** Box and whiskers plot showing comparison of Ct values for two reference genes *EF2* and *ACTB* in SAT and VAT adipose tissues of lean (BMI < 28; n = 9 for SAT and n = 7 for VAT; and obese (BMI > 30; *MCPIP1*: n = 12 for SAT and n = 17 for VAT) subjects estimated by Q-RT-PCR (TIF 745 kb)

Supplementary Fig. 2 GO enrichment analysis of differentially expressed genes in 3T3-L1 adipocytes overexpressing wild-type or mutant MCPIP1 at day 2 of differentiation. **a** GO biological terms enriched for differentially expressed genes in _WT_MCPIP1 vs. Control and **b**_WT_MCPIP1 vs. _D141N_MCPIP1 adipocytes. Scale is the –log10 (p-value) of the enrichment score. Red bars indicate upregulated genes and blue bars downregulated genes (p-value < 0.05; fold change ≥ 1.5) (TIF 11760 kb)

Supplementary Fig. 3 Correlation analysis of RNA-seq data with Q-RT-PCR analysis between adipocytes expressing _WT_MCPIP1 and _D141N_MCPIP1 or Control cells. Statistical significance was determined with Pearson correlation (TIF 192 kb)

Supplementary Fig. 4 KEGG pathways enrichment analysis of proteins altered in 3T3-L1 adipocytes overexpressing wild-type or mutant MCPIP1 at day 4 of differentiation. **a** KEGG pathways differentially altered in _WT_MCPIP1 vs. Control and **b**_WT_MCPIP1 vs. _D141N_MCPIP1 adipocytes. Graphs present the number of the annotated proteins in each pathway. Scale is the –log10(p-value) of the enrichment score. Red bars indicate upregulated pathways and blue bars downregulated pathways (p-value < 0.05; fold change ≥ 1.5) (TIF 5036 kb)

Supplementary Fig. 5 Comparison of transcriptomic and proteomic data in 3T3-L1 adipocytes expressing wild-type or mutant MCPIP1. **a** Comparison of biological processes identified by DAVID for transcriptome data (version 6.8; https://david.ncifcrf.gov/home.jsp) and for proteome data estimated by IPA analysis in _WT_MCPIP1 vs. Control and **b**_WT_MCPIP1 vs. _D141N_MCPIP1 groups. Red and blue colors indicate predicted upregulation/activation (*z*-score > 1) and downregulation/inhibition (*z*-score < -1) of biological processes, respectively (TIF 11999 kb)

Supplementary Fig. 6 Comparison of RNA-Seq and proteomics data in 3T3-L1 adipocytes expressing wild type or mutant MCPIP1. **a** Proportions of transcripts and proteins annotated to KEGG Pathways predicted as downregulated in _WT_MCPIP1 vs. Control and **b**_WT_MCPIP1 vs. _D141N_MCPIP1 and **c** upregulated in _WT_MCPIP1 vs. Control group (p-value < 0.05). Scale is the –log10 (p-value) of the enrichment score. White color and crosshatched bars indicate number of the annotated transcripts and proteins, respectively (TIF 1481 kb)

Supplementary Fig. 7 MCPIP1 decreases levels of PPARγ and C/EBPα in 3T3-L1 adipocytes **a** Representative Western blot for MCPIP1, PPARγ and C/EBPα protein levels from total cell lysates of _WT_MCPIP1, _D141N_MCPIP1 and adipocytes with an empty vector (Control) at different time points of adipognesis (day 0, 2, 4 and 8). **b** Densitometric quantification of PPARγ (n = 3 for day 2 and 4; n = 4 for day 8) and **c** C/EBPα (n = 3 for day 2; n = 4 for day 4 and 8) protein levels normalized to β-Actin. Data are presented as fold change (normalized to control cells) and as mean ± SD. Statistical significance was determined with one-way ANOVA. * *p* < 0.05; *** *p* < 0.001 **b** Oil O Red staining of lipid droplets accumulated by 3T3-L1 adipocytes overexpressed wild-type or mutant MCPIP1 and control cells at day 2, 4 and 8 of adipogenic differentiation. The pictures represent data from one of three independent experiments. Scale bar represents 100 µm (TIF 11677 kb)
