# Supplementary material for: Integrative genomics reveal a role for MCPIP1 in adipogenesis and adipocyte metabolism
Source: Cell Mol Life Sci. 2019 Dec 31;77(23):4899–919. doi: 10.1007/s00018-019-03434-5 (PMC7658075; doi:10.1007/s00018-019-03434-5)
Supplement: Supplementary file 1 — Supplementary file1 (DOCX 13 kb) [file 18_2019_3434_MOESM1_ESM.docx]

Supplementary Table 1. Patient’s characteristic.

| **Variable** | **Lean** | **Obese** |
| --- | --- | --- |
| No. of subjects | 9 | 19 |
| Sex Male | 6 | 8 |
| Female | 3 | 11 |
| Age, median (± SD) years | 44 (15) | 44 (11) |
| BMI, median (± SD) kg/m^2^ | 24.7 (3.6) | 47.5 (7.6) |
| No. of T2D subjects | 1 | 11 |

BMI, body mass index; T2D, Type 2 diabetes
