# Supplementary material for: Integrative genomics reveal a role for MCPIP1 in adipogenesis and adipocyte metabolism
Source: Cell Mol Life Sci. 2019 Dec 31;77(23):4899–919. doi: 10.1007/s00018-019-03434-5 (PMC7658075; doi:10.1007/s00018-019-03434-5)
Supplement: Supplementary file 3 — Supplementary file3 (DOCX 16 kb) [file 18_2019_3434_MOESM3_ESM.docx]

Supplementary Table 3. Real-time PCR primer sequences. All primer sequences were designed according to GenBank sequences.

| **Gene name** | **NCBI accession no.** | **Primer sequence** |
| --- | --- | --- |
| Zinc finger CCCH-type containing 12A (ZC3H12A) | NM_025079.3 | GGAAGCAGCCGTGTCCCTATG  TCCAGGCTGCACTGCTCACTC |
| Interleukin 6 (IL6) | NM_000600 | GTGAAAGCAGCAAAGAGGCA  TCACCAGGCAAGTCTCCTCA |
| C-C motif chemokine ligand 2 (MCP-1) | NM_002982.4 | CTTCTGTGCCTGCTGCTCATAGC  CCAGGTGGTCCATGGAATCCTG |
| CD68 molecule (CD68) | NM_001251.3 | AGGTCCAGGGAAGCTGTGAG  GAATGTCCACTGTGCTGCGT |
| solute carrier family 2 member 4 (SLC2A4) | NM_001042.3 | CGACCAGCATCTTCGAGACAG  CACCAACAACACCGAGACC |
| Eukaryotic translation elongation factor 2 (Eef2) | NM_007907.2 | GACATCACCAAGGGTGTGCAG  TTCAGCACACTGGCATAGAGGC |
| Diacylglycerol O-acyltransferase 2 (Dgat2) | NM_026384.3 | GCCGATGGGTCCAGAAGAAG  CGATGTCTTTCTGGGTCGGG |
| Solute carrier family 2 member 4 (Slc2a4) | NM_009204.2 | GCCCCACAGAAGGTGATTGA  AGAGAGCCCAGAGCGTAGTG |
| Stearoyl-CoA desaturase (Scd1) | NM_009127.4 | GAAAGTGAGGCGAGCAACTG  GTGGTCGTGTAAGAACTGGAGA |
| Sterol regulatory element-binding protein 1 (Srebp1) | NM_011480.4 | GGAACTTTTCCTTAACGTGGGC  ATGAGCTGGAGCATGTCTTCG |
| Signal transducer and activator of transcription 5A (Stat5a) | NM_011488.3 | ACTCTTCGGGATGGGGACTAT  GGTGGAGGCTGTTACTTCTAAAC |
| Mannosyl (Alpha-1,3-)-Glycoprotein Beta-1,2-N-Acetylglucosaminyltransferase (Mgat1) | NM_010794.3 | CCTGTTTCTTCTCCTGAGCCC  GGCGCGTGTTACATCCCTTC |
| Dual specificity protein phosphatase 4 (Dusp4) | NM_176933.4 | GCCTGCTTAAAGGTGGCTATG  TTGGTGCTGGGAGGTACAGG |
| Solute carrier family 25 member 10 (Slc25a10) | NM_013770.2 | ACGCAACTACTCTCATGCCC  AAAACACCCTGGTACTCGCC |
| Elongation of very long chain fatty acids protein 1 (Elovl1) | NM_001039176.2 | GGGCAGGAGTCTCAAAGAGC  AGGCCCAAGCGATAGGATGA |
| CCAAT enhancer binding protein alpha (Cebpa) | NM_007678.3 | GCCTTCAACGACGAGTTCCT  CCGGGTAGTCAAAGTCACCG |
| Fibronectin leucine rich transmembrane protein 2 (Flrt2) | NM_201518.4 | GGAGACAAGGCTGCCAGATTA  AAGCAAAGCGTGATGCGAAGT |
| GLI family zinc finger 1 (Gli1) | NM_010296.2 | CAGCATGGGAACAGAAGGACT  GAAAGGGGCGAGATGGAGAG |
| Lipoprotein lipase (Lpl) | NM_008509.2 | CTTTCACTCGGATCCTCTCG  AGGTGGACATCGGAGAACTG |
| TBC1 domain family member 4 (Tbc1d4) | NM_001081278.2 | CACGACCTCACCTACTTTGCC  ACTGATCACATCTGGAACCTGG |
| Actin beta (ACTB) | NM_001101.5 | CAAGAGATGGCCACGGCTGCTT  CAGGTCTTTGCGGATGTCCACG |
